# Diagnosis of Swimming Induced Pulmonary Edema—A Review

**DOI:** 10.3389/fphys.2017.00652

**Published:** 2017-08-31

**Authors:** Hannes Grünig, Pantelis T. Nikolaidis, Richard E. Moon, Beat Knechtle

**Affiliations:** ^1^Institut für Radiologie, Luzerner Kantonsspital Luzern, Switzerland; ^2^Exercise Physiology Laboratory Nikaia, Greece; ^3^Center for Hyperbaric Medicine and Environmental Physiology, Department of Anesthesiology, Duke University Medical Center Durham, NC, United States; ^4^Gesundheitszentrum St. Gallen St. Gallen, Switzerland; ^5^Institute of Primary Care, University of Zurich Zurich, Switzerland

**Keywords:** athlete, breathing, diving, radiologic examination, swimmer, water sports

## Abstract

Swimming induced pulmonary edema (SIPE) is a complication that can occur during exercise with the possibility of misdiagnosis and can quickly become life threatening; however, medical literature infrequently describes SIPE. Therefore, the aim of this review was to analyse all individual cases diagnosed with SIPE as reported in scientific sources, with an emphasis on the diagnostic pathways and the key facts resulting in its diagnosis. Due to a multifactorial and complicated pathophysiology, the diagnosis could be difficult. Based on the actual literature, we try to point out important findings regarding history, conditions, clinical findings, and diagnostic testing helping to confirm the diagnosis of SIPE. Thirty-eight cases from seventeen articles reporting the diagnosis of SIPE were selected. We found remarkable differences in the individual described diagnostic pathways. A total of 100% of the cases suffered from an acute onset of breathing problems, occasionally accompanied by hemoptysis. A total of 73% showed initial hypoxemia. In most of the cases (89%), an initial chest X-Ray or chest CT was available, of which one-third (71%) showed radiological signs of pulmonary edema. The majority of the cases (82%) experienced a rapid resolution of symptoms within 48 h, the diagnostic hallmark of SIPE. Due to a foreseeable increase in participation in swimming competitions and endurance competitions with a swimming component, diagnosis of SIPE will be important, especially for medical teams caring for these athletes.

## Introduction

Swimming induced pulmonary edema/oedema (SIPE/SIPO) is drawing more and more attention due to the increasing quantity of pertinent information about water sports. However, it is infrequently described in the medical literature. The cases of pulmonary edema during different sport activities are inconsistently categorized as exercised-induced pulmonary edema (EIPE) and swimming induced pulmonary edema (SIPE). The cases of SIPE thereby often include every case of pulmonary edema during water sport activity without a differentiation between surface swimming, snorkeling, scuba diving or breath-hold diving. Typical symptoms include dyspnoea, chest pain or chest tightness, cough, and occasionally hemoptysis. The diagnostic criteria vary and the diagnosis is sometimes suggested by self-reported symptoms of the patient.

Estimates of the prevalence of SIPE vary considerably. Among combat swimmer trainees during 2.4–3.6 km open sea swimming trials the prevalence has been reported to be 1.8–60%, depending upon the severity (Shupak et al., 2000). The prevalence of symptoms highly suggestive of SIPE among triathletes has been reported as 1.4% (Miller et al., 2010).

Fatal cases associated with SIPE have been reported regarding a snorkeler and a scuba diver (Smart et al., 2014). Predisposing factors for SIPE include cardiopulmonary disease (especially hypertension) and pulmonary hypertension (Peacher et al., 2015). Risk factors include cold water, exercise, elevated negative inspiratory pressure, age, female sex, and emotional stress (Gempp et al., 2014).

The pathophysiology of SIPE is poorly understood. It seems to occur mostly in apparently healthy individuals. Cardiopulmonary disease could be a predisposing factor for developing pulmonary edema during swimming or diving (Peacher et al., 2015). Ludwig et al. (2004) performed acute and post recovery bronchoalveolar lavage (BAL) in a subject suffering from SIPE and found no evidence of an underlying infection. Immersion causes centralization of blood to the heart, leading to an increased central blood volume (Arborelius et al., 1972). Lange et al. (1974) showed an increase in heart volume when immersing a standing subject. Exercise in cold water can augment this effect (Wester et al., 2009). Zarvorsky (2007) reviewed literature and showed that pulmonary edema can also occur by exercise alone, especially during maximal effort exercise. In a few published cases, unilateral pulmonary edema occurred during swimming, suggesting an underlying hemodynamic mechanism (Mahon et al., 2002; Lund et al., 2003).

More recent studies suggest a right and left ventricular stroke volume mismatch and raised mean pulmonary arterial pressure and pulmonary arterial wedge pressure (Casey et al., 2014; Moon et al., 2016b). Pharmaceutical agents have been examined for their effectiveness in SIPE prevention. Moon et al. (2016b) observed a higher mean pulmonary arterial pressure and pulmonary arterial wedge pressure in individuals with a history of SIPE during submerged exercise in cold water and showed that these pressures can be reduced with sildenafil.

While most SIPE cases in the years after the first documented cases of SIPE in the 80 s occurred mostly in the context of military training (Weiler-Ravell et al., 1995), it seems to occur more and more during various swimming competitions or sport events including a swimming section. SIPE is a complication that can occur during exercise with the possibility of misdiagnosis and can quickly become life threatening. Between October 2008 and November 2015; Moon et al. (2016a) identified 42 deaths of triathletes during a swim. Of these, 23 *post-mortem* reports were obtained. They found evidence in these *post-mortem* examinations of SIPE susceptibility markers (LVH) in excess of the prevalence expected among healthy triathletes.

The following review provides an overview of published individual cases with the diagnosis of SIPE. We focus on the diagnostic pathways and the documentation in the published cases. The point of interest is the key factors that resulted in the diagnosis of pulmonary edema.

## Materials and methods

### Search methods

Literature research was performed in PubMed, Scopus and Google Scholar using the key words “swimming pulmonary edema/oedema” and “immersion pulmonary edema/oedema.” Searches were limited to articles published between January 1989 and May 2016.

### Study selection

Articles were assessed for relevance to the topic. We included every publication with at least one individual case of a reported pulmonary edema associated with swimming activities. There were no restrictions on language of publication. We excluded publications with reported pulmonary edema associated with diving activities including snorkeling and scuba diving.

### Research outcome

Thirty-nine articles were identified with a total number of 361 cases. Twenty-two articles were excluded due to the association with diving activities or the absence of specific case-descriptions. Seventeen articles remained, reporting a total number of 38 individual documented cases of pulmonary edema (Table 1). These cases included 10 women (26%) and 28 men (74%). Their age ranged from 18 to 60 years. Thirty-seven cases were associated with swimming activities, and one case suffered pulmonary edema during aqua jogging.

**Table 1:** The cases presented in detail.

**Number**	**Age**	**Gender**	**Race ore activity**	**Medical history**	**Water T °C**	**Wetsuit**	**Initial symptoms**	**Haemoptysis?**	**Pulmonary auscultation**	**InitialO_2_%**	**Therapy**	**Pulmonary edema on initial chest X-Ray or computer tomography (CT)?**	**Resolution of symptoms in 48 h? (and exact time specification)**	**Normal follow up X-ray**	**Remarks**	**References**
1	18	m	Military fitness training	n/a	23°C	No	Cough	Yes	n/a	95%	Supportive O_2_	Yes Infiltrates on chest X-Ray	Yes (Overnight) yes	Yes	Hydration with 5 L water	Weiler-Ravell et al., 1995
2	18	m	Military fitness training	n/a	23°C	No	Cough	Yes	n/a	90%	Supportive O_2_	No	Yes (Overnight)	n/a	Hydration with 5 L water	Weiler-Ravell et al., 1995
3	19	m	Military fitness training	n/a	23°C	No	Cough	Yes	n/a	98%	Supportive O_2_	No	Yes (Overnight)	n/a	Hydration with 5 L water	Weiler-Ravell et al., 1995
4	18	m	Military fitness training	n/a	23°C	No	Cough	Yes	n/a	84%	Supportive O_2_	No	yes (Overnight)	n/a	Hydration with 5L water	Weiler-Ravell et al., 1995
5	18	m	Military fitness training	n/a	23°C	No	Cough	Yes	n/a	81%	Supportive O_2_	No	Yes (Overnight)	n/a	Hydration with 5 L water	Weiler-Ravell et al., 1995
6	19	m	Military fitness training	n/a	23°C	No	Cough	Yes	n/a	88%	Supportive O_2_	No	Yes (Overnight)	n/a	Hydration with 5 L water	Weiler-Ravell et al., 1995
7	18	m	Military fitness training	n/a	23°C	No	Cough	Yes	n/a	96%	Supportive O_2_	No	Yes (Overnight)	n/a	Hydration with 5L water	Weiler-Ravell et al., 1995
8	18	m	Military fitness training	n/a	23°C	No	Cough	Yes	n/a	87%	Supportive O_2_	Yes Infiltrates on chest X-Ray	Yes (Overnight)	Yes	Hydration with 5 L water	Weiler-Ravell et al., 1995
9	39	f	Swim across lake zürich	None	20.6°C	n/a	Shortness of breath, cough	No	n/a	n/a	None	n/a	Yes (Within a few hours)	n/a		Pons et al., 1995
10	23	m	Swim across lake zürich	None	20.6°C	n/a	Shortness of breath, cough	Yes	n/a	n/a	n/a	Yes Pulmonary edema	Yes (Pulmonary oedema cleared after 8 h)	n/a		Pons et al., 1995
11	22 -28	m	2 Mile Swim	None	17°C	Yes	Severe dyspnoea, non-productive cough	No	Unilateral crackles	Hypoxemia	Supportive O_2_β_2_-agonist	Yes Unilateral Kerley-B, cephalization, airspace consolidation	Yes (Yes, within 24–48 h)	Yes		Mahon et al., 2002
12	22 -28	m	2 Mile Swim	None	17°C	Yes	Severe dyspnoea, non-productive cough	No	Unilateral crackles	Hypoxemia	Supportive O_2_β_2_-agonist	Yes Unilateral Kerley-B, cephalization, airspace consolidation	Yes (Yes, within 24–48 h)	Yes		Mahon et al., 2002
13	22 -28	m	2 Mile Swim	None	17°C	Yes	Severe dyspnoea, non-productive cough	No	Unilateral crackles	Hypoxemia	Supportive O_2_β_2_-agonist	Yes Unilateral Kerley-B, cephalization, airspace consolidation	Yes (Yes, within 24–48 h)	Yes		Mahon et al., 2002
14	21	m	2 Mile Ocean Swim	None	19.2°C	Yes	Shortness of breath, cough, and confusion	No	Expiratory wheezes throughout the right lung fields	85%	Supportive O_2_ β_2_-agonist	Yes Right lower lobe infiltrate, Kerley-B left side	Yes (on Day 2) (yes)	Yes		Lund et al., 2003
15	25	m	2 Mile Ocean Swim	Pneumonia 6 months ago, left renal agenesis	19.2°C	Yes	Shortness of breath, cough, confusion, dyspnoea	No	Expiratory wheezes throughout the right lung fields.	90%	Supportive O_2_β_2_-agonist	Yes Right lower lobe infiltrate	No	No X-Ray on Day 2 almost normal, on Day 5 normal		Lund et al., 2003
16	27	m	2 Mile Ocean Swim	None	19.2°C	Yes	Dyspnoea, shortness of breath, cough	Yes	Expiratory wheezes in the right lung base	96%	Supportive O_2_β_2_-agonist	No	Yes (after 5 h)	n/a		Lund et al., 2003
17	36	m	Training for ironman	Type 1 diabetes insulin	n/a	Yes	Shortness of breath, cough	Yes	Crackles on the right side	94%	None	No	Yes (He improved quickly on the ward and was asymptomatic)	n/a		Biswas et al., 2004
18	38	f	Training	n/a protein supplement	15°C	Yes	Chest constriction, wheezing, dyspnoea	Yes	Diffuse wheezing	72%	Supportive O_2_diuretics	Yes Alveolar infiltrates	n/a (Follow up X-Ray on day 5 was normal)	n/a		Deady et al., 2006
19	43	m	Aqua jogging	n/a	20°C	No	Chest pressure, shortness of breath	Yes	n/a	n/a	Supportive O_2_	Yes Bilateral infiltrates and Kerley-B Lines	Yes (Patient recovered within 24 h)	n/a	Aqua jogging	Wenger and Russi, 2007
20	54	f	Training	Hypertension	22°C	n/a	Shortness of breath	No	n/a	64%	Supportive O_2_CPAP diuretics	n/a	n/a	n/a		Beinart et al., 2007
21	23	f	Swiss gigathlon 2007	none	n/a	n/a	Shortness of breath	Yes	Fine bilateral crackles	73%	Antibiotics	Yes Bilateral alveolar infiltrates	yes (next Day) (Yes, normal X-Ray)	Yes		Noti et al., 2009
22	37	m	Swiss gigathlon 2007	Mild mitral valve insufficiency	14°C	n/a	Shortness of breath	No	Bilateral rales	60%	Supportive O_2_CPAP	Yes Bilateral alveolar infiltrates	yes (Next Day)	No only considerably reduction of infiltrates		Bloch et al., 2009
23	28	m	Training	n/a	‘Cold’	n/a	Cough, dyspnoea	Yes	n/a	89%	Supportive O_2_β_2_-agonist	Yes Bibasilar interstitial and airspace process	Yes (<48 h)	Yes		Shearer and Mahon, 2009
24	31	m	Training	n/a	“Cold”	n/a	Cough, dyspnoea	Yes	n/a	97%	Supportive O_2_β_2_-agonist	Yes Interstitial prominence in the lung base	Yes (<48 h)	Yes		Shearer and Mahon, 2009
25	23	m	Training	n/a	“Cold”	n/a	Cough, dyspnoea	Yes	n/a	99%	Supportive O_2_β_2_-agonist	Yes Mixed interstitial and airspace process	Yes (<48 h)	Yes		Shearer and Mahon, 2009
26	25	m	Training	n/a	“Cold”	n/a	Cough, dyspnoea	Yes	n/a	87%	Supportive O_2_β_2_-agonist	Yes Mixed interstitial and airspace process	Yes (<48 h)	Yes		Shearer and Mahon, 2009
27	29	m	Training	n/a	“Cold”	n/a	Cough, dyspnoea	Yes	n/a	87%	Supportive O_2_β_2_-agonist	Yes Bibasilar diffuse interstitial and airspace process	Yes (<48 h)	Yes		Shearer and Mahon, 2009
28	27	m	Training	n/a	“Cold”	n/a	Cough, dyspnoea	Yes	n/a	n/a	Supportive O_2_β_2_-agonist	Yes Linear opacities in right lung base	Yes (<48 h)	Yes		Shearer and Mahon, 2009
29	43	f	Military training	n/a	16°C	Yes	Shortness of breath, chest pain	Yes	Crackles throughout all lobes	n/a	None	n/a	Yes	Yes		Knutson, 2010
30	58	f	Training for triathlon	n/a	15°C	n/a	Shortness of breath, cough	Yes	Crackles and rales	94%	None	Yes Patchy opacity left upper lobe anterior segment	Yes (Next Day)	Yes		Carter and Koehle, 2011
31	45	f	Training	Asthma, allergies	18°C	Yes	Shortness of breath, cough	No	Rales	85%	n/a	Yes Bilateral ground glass predominately on the lower right side	n/a (Follow-up X-Ray after 2 h showed significant improvement with residual slight ground glass abnormalities)	n/a		Carter and Koehle, 2011
32	43	f	Ironman	none	22°C	Yes	Shortness of breath, cough	Yes	Rales	76%	Supportive O_2_Diuretics	Yes CR confirmed pulmonary edema	n/a	n/a		Carter and Koehle, 2011
33	26	f	Attempting to swim the English channel	n/a	n/a	n/a	Breathlessness, cough	Yes	Scattered crepitation	89%	Supportive O_2_diuretics antibiotics	Yes Bilateral airspace shadowing	Yes (Improved clinically 2 days later. X-Ray only shown without description)	n/a	Symptoms 4 h after the swim	North and Mansfield, 2013
34	57	m	Triathlon	Hypertension hypercholesterinaemia perindopril, avastatin	12.4–19.1°C	n/a	Dyspnoea	Yes	Bilateral wheezes	84%	Supportive O_2_β_2_-agonist diuretics	n/a	n/a	n/a		Ma and Dutch, 2013
35	60	m	Ironman	none	13°C	Yes	Dyspnoea	No	Fine bilateral crackles	86%	Diuretics β_2_-agonist nitroglycerine	No Chest X-ray normal	Yes (In a view hours)	n/a		Casey et al., 2014
36	55	f	Training	none	n/a	n/a	Shortness of breath	Yes	End expiratory crackles	95%	β_2_-agonist	No Chest X-ray normal	Yes (She was discharged home the same day)	n/a		Casey et al., 2014
37	38	m	Triathlon Race	none	21.4°C	n/a	Difficulty in breathing	No	Bilateral wheezing	82%	Prednisolon Diuretics	Yes Bilateral ground glass opacity in the peripheral lung (CT-scan)	Yes (All symptoms of pulmonary edema resolved within 4.5 h)	n/a		Yamanashi et al., 2015
38	57	m	Swimming	none	n/a	n/a	Dyspnoea, chills	No	n/a	75%	n/a	Yes diffuse consolidation	n/a	n/a		Yamanashi et al., 2015

## Results

Wilmshurst et al. (1989) published 11 cases of divers who developed pulmonary edema during scuba diving in cold water (temperature below 12°C). Two of them also had an episode while swimming at the surface. These two cases were the first documentation of pulmonary edema during surface swimming activities. A physician or a cardiologist at the hospital without a detailed description of the diagnostic details made the diagnosis of cold induced pulmonary edema. In some individuals, the symptoms resolved quickly when they left the water, so the diagnosis was made simply by the history, not with a physical examination.

Pons et al. (1995) published two cases of pulmonary edema during diving activity, as well as two more detailed cases of pulmonary edema during surface swimming. One of these patients developed the first episode of pulmonary edema during a dive to 25 m. The diagnosis was established in hospital by physician examination and by chest X-ray. He then suffered another three episodes of similar symptoms during surface swimming, but received no physical examination or chest X-ray. The second individual case developed pulmonary edema during swimming across Lake Zürich with the symptoms of cough, hemoptysis and shortness of breath. The diagnosis of pulmonary edema was made in hospital by clinical results and by chest X-ray. The pulmonary edema cleared within 8 h with no report of the physical findings or a follow-up chest X-ray.

Weiler-Ravell et al. (1995) published eight individual cases of pulmonary edema induced by strenuous swimming. All of them developed cough and hemoptysis, and they measured reduced oxygen saturation. Two of them had also infiltrates on their chest X-Ray. Attention should be paid to the over-hydration (5 L of water) preceding the swimming activity of all eight swimmers. There was also a quick resolution of the signs and symptoms overnight.

Shupak et al. (2000) published a field study where they studied 35 young men during 2 months participating in a fitness-training programme with repetitive swimming trials in the open sea. Of these, 60% experienced an episode of SIPE. The diagnosis of SIPE was made when the swimmers reported shortness of breath accompanied by coughing without prior seawater aspiration. In most cases of SIPE, shortness of breath, and coughing were accompanied by hemoptysis and basilar rales on chest auscultation. They also measured O_2_ saturation immediately after the swimming trial, where they found a significantly reduced saturation compared with asymptomatic participants.

Mahon et al. (2002) reported three cases of young combat swimmers who suffered from severe dyspnoea, non-productive cough, hypoxemia, unilateral crackles, and unilateral signs of pulmonary edema on their chest X-ray. In all three swimmers, hypoxemia resolved within 12 h and the findings on their chest X-Ray within 24–48 h. Another three cases with more detailed reports were published by Lund et al. (2003) where they again observed the rapid normalization of the symptoms caused by pulmonary edema within 24–48 h, specifically dyspnoea, cough, occasionally hemoptysis, hypoxemia, and chest radiograph with signs of pulmonary edema.

Shortly after that, Adir et al. (2004) published a series of 70 summarized cases of SIPE in healthy young men. SIPE was diagnosed when severe shortness of breath and coughing were reported during or after swimming, and evidence of pulmonary edema was found on physical examination, specifically when bilateral inspiratory crackles which failed to clear with deep inspiration were found. A basic criterion was the absence of water-aspiration. Results of chest radiographs obtained 12–18 h after the onsets of symptoms were normal in all cases. They also found a significantly lower O_2_-saturation in all subjects (average 88.4%).

In the same year, Biswas et al. (2004) reported one individual case of swimming induced pulmonary edema precipitated by cold water swimming while wearing a wet suit. When the symptoms began, he loosened his wet suit to let the cold water in, but the breathing worsened, and after half a mile, he started to cough up blood stained sputum. After administration of salbutamol, he visited a general practitioner who heard crackles on the right side. On admission to the hospital about 8–9 h after the incident, he improved quickly without any pathology on the chest X-ray and normal oxygen saturation (94%). Except for type 1 diabetes, his medical history was unremarkable.

SIPE was also the diagnosis in one individual case published in 2004 by Yoder and Viera (2004) whereas a young rescue swimmer suffered from chest pain, shortness of breath, cough with pink sputum, an initial oxygen saturation of 70% and bilateral rhonchi on auscultation after performing a free dive to 12 feet (3.65 m). The initial X-ray showed central infiltrates which resolved on the follow-up chest X-ray several hours later.

Another two individual reported cases of pulmonary edema during diving activity were published by Peacher et al. (2015). Symptoms of severe pulmonary edema developed upon diving to 30 feet of seawater (9.13 m) and 25 feet of sea water (7.61 m) respectively.

Koehle et al. (2005) analyzed 60 cases of pulmonary edema during water activities (i.e., breath-hold diving, scuba diving, surface swimming) most of which were scuba divers. They found the most common symptoms to be cough, dyspnoea, and hemoptysis. The most common investigation was a chest X-ray showing the typical signs of pulmonary edema. Symptoms mostly resolved within 24 h.

In 2006, Deady et al. (2006) published a diagnostic challenge with several possible solutions. The patient developed dyspnoea, chest constriction and wheezing after swimming 500 m in cold water while wearing a wetsuit. In the emergency room, they found hypoxemia (72%), and bilateral infiltrates on the chest X-ray. Symptoms resolved quickly without an exact time specification and a control chest X-ray 5 days later was normal. The correct answer to the challenge was SIPE.

Ludwig et al. (2006) tried to find differences in cardiopulmonary function between individuals with a history of SIPE and controls. They were the first who described in detail four diagnostic criteria of SIPE. These included acute onset of dyspnoea or hemoptysis during or immediately after swimming, hypoxemia, defined by oxygen saturation <92% or an alveolar-arterial oxygen gradient of >30 mmHg, radiographic opacities consistent with an alveolar filling process and/or interstitial pulmonary edema that resolve within 48 h and no history of water aspiration, laryngospasm, or preceding infections.

Wenger and Russi (2007) published the only case of an aqua jogging-induced pulmonary edema. The diagnosis was made by the combination of chest-pressure, shortness of breath, expectoration of bloody froth and signs of pulmonary edema on the chest X-ray and chest CT. Symptoms resolved within 24 h. A follow up radiograph was not mentioned.

Beinart et al. (2007) reported one case of a middle-aged woman with a history of hypertension who developed shortness of breath after swimming. Oxygen saturation was relatively low (64%) and she was admitted to emergency care where they found elevated troponin I and left and right ventricular dysfunction by echocardiography. Symptoms resolved following supportive therapy without a precise time specification. A control-echocardiography on day five after hospitalization was normal.

A detailed report of SIPE from two participants of the “Swiss Gigathlon” were published in 2009 (Bloch et al., 2009; Noti et al., 2009). Both suffered from shortness of breath, one additional from cough with hemoptysis. Both showed diminished oxygen saturation (73 and 60% respectively) and typical signs of pulmonary edema on the chest X-ray. All symptoms resolved completely in both individuals and a control chest X-ray the next day was normal.

In the same year, Shearer and Mahon (2009) analyzed six basic underwater demolition/SEAL recruits with the diagnosis of SIPE for their brain natriuretic peptide level (BNP-level). They described their case definition of SIPE and made the diagnosis by the presence of hypoxemia occurring during or immediately after a swimming event, a demonstrable chest X-ray abnormality, and improvement or resolution of these abnormalities in <48 h. Of importance was the absence of pulmonary infection and no history of breathing against a closed glottis or aspiration.

Miller et al. (2010) published a large study about SIPE in community triathletes in 2010. The study was based on a questionnaire that reached a big number of triathletes in the US. Their case definition of SIPE was limited to self-reported symptoms of coughing up pink froth or blood-tinged secretions. Amongst the study population of 1,400 triathletes, 20 reported having experienced the symptoms described, which correlates to a prevalence of 1.4%. With 11 additional cases, they analyzed different risk factors for SIPE and found a statistically significant correlation with history of hypertension, consumption of fish oil supplements, wetsuit use and long course distance (half-Ironman or greater).

In the same year, Knutson (2010) reported on a female military major training for a swimming competition who developed a very productive cough after finishing the training, but continued with a 35 km bike ride. Three day later, during the next swim, the symptoms worsened with an acute onset, accompanied by chest pain with palpitations. The symptoms worsened again after loosening the wetsuit to let the cold (16°C) water in. She developed bloodstained sputum and coughed up pink froth. The symptoms resolved within 6 h. The chest X-ray 2 days later was normal.

One year later, Carter and Koehle (2011) published three cases of female triathletes who developed SIPE, two during their training, and one during swimming in a half-Ironman. All developed shortness of breath and cough, one of them with hemoptysis. Two had significantly low oxygen saturations (85, 76% respectively) and all demonstrated a pathologic initial chest X-ray. Two of them received a follow up chest X-ray; one was normal (after 1 day), the second showed significant improvement (after 2 h). There was no reported follow up on the third triathlete. Interestingly, two of them had four more episodes of SIPE, all in cold water.

North and Mansfield (2013) published a detailed report about a young female subject who developed pulmonary edema after attempting to swim the English Channel in 2013. Four hour after finishing the swim, she developed breathlessness associated with a cough productive of pink sputum. Her initial oxygen saturation was 89% and the chest X-ray demonstrated bilateral airspace shadowing. The symptoms improved within 2 days.

A case series of five triathlon-participants in Victoria, Australia in 2013 with shortness of breath and hemoptysis was published by Ma and Dutch (2013). They diagnosed exercise induced pulmonary edema in four of them, while one participant developed the symptoms after the swimming part. His initial oxygen saturation was 84% and immersion pulmonary edema was suspected. The patient was transported to the hospital for further care. Follow-up information was not available.

Casey et al. (2014) published two cases of SIPE in two triathletes, one taking part in an Ironman triathlon, and the other one during a training swim. Both developed breathing problems, one of them with hemoptysis, the other one with decreased oxygen saturation of 86%. Both showed a normal initial chest X-ray. The first of them recovered from the initial symptoms in a few hours, and the second already had a clear chest upon arrival to the medical admissions unit.

Also Yamanashi et al. (2015) published a study of a group of five triathletes with pulmonary edema at a triathlon race, two of them with onset of the symptoms during or directly after the swimming part. One of them is reported in detail as having difficulty in breathing while swimming in relatively warm water (21.4°C). A respiratory examination showed bilateral wheezing and the initial oxygen saturation was 82%. He was delivered to the hospital, where a thoracic computer tomography (CT) showed bilateral ground glass opacity. All his symptoms of pulmonary edema resolved within 4.5 h. A follow up CT-scan or chest X-ray is not reported. The second case is published in table form, and shows that the patient also developed dyspnoea and the chills after swimming for 30 min. The initial oxygen saturation was 75% and a chest X-ray showed diffuse consolidation. His admission period was 4 days without a report of detailed follow up examinations.

## Discussion

Since the first reported case of SIPE in 1989, the phenomenon of sudden development of pulmonary edema during strenuous swimming has been increasingly recognized as a discrete diagnosis. In this review, we summarized 38 individual cases with the diagnosis of SIPE available in scientific sources with an analysis of the diagnostic pathways (see Table 1).

### Diagnosis of SIPE

#### Initial symptoms and conditions

SIPE is characterized by an acute onset of breathing problems caused by accumulation of fluid in the lung extravascular space, induced by immersion, usually but not always in cold water, and intense physical activity. All of the 38 cases (100%) developed their symptoms during physical activity (e.g., military fitness training, lake crossing, training or aqua jogging) six of them (16%) during participation on races. Mean temperature was 19.6°C (ranging from 23° to 13°C).

Main initial reported symptoms were dyspnoea / shortness of breath (79%) and/or cough (71%). Three (8%) suffered from chest tightness or chest pain and two (5%) from confusion. Haemoptysis was also a main discovery reported in 26 cases (68%), approximately similar to the observations made by Adir et al. (2004), where they observed hemoptysis in 55.7% subjects diagnosed with SIPE. Athletes can also develop hemoptysis during running (Kim et al., 2015; Kruvait et al., 2016). Hopkins et al. (1997) studied a group of elite cyclists and found higher red blood concentration in the BAL after intense exercise compared to normal subjects without exercise. Interestingly, they found no difference between the two groups after submaximal exercise (Hopkins et al., 1998), suggesting that maximal stress to the blood gas barrier is necessary to produce hemoptysis. The fact that 68% of the cases we summarized developed hemoptysis might confirm the hypothesis that this level of stress is often reached by athletes during submaximal to maximal exercise in combination with immersion.

If considered individually, these symptoms are not very specific and could be caused by other conditions such as water aspiration, infections of the respiratory tract or an acute asthma attack. Nevertheless, if they occur in combination with the conditions mentioned above, SIPE is a possibility. Auscultation was reported in 19 of the 38 cases wherein all (100%) showed abnormalities, mostly crackles (47%) or wheezing (32%) suggesting an airway process.

#### Oxygen saturation

Desaturation could be a sign of pulmonary edema where fluid overload in the lungs lead to an intrapulmonary shunt with low ventilation/perfusion ratio. In 33 cases (87%), initial oxygen saturation was available, wherein 24 (73%) showed hypoxemia.

Nevertheless, reduced oxygen saturation is nonspecific. Other pathologic factors like infections, emphysema or interstitial lung disease could lead to hypoxemia occurring only during exercise. These conditions are impairing the oxygen transport across the alveolar-capillary membrane. When pulmonary capillary transit time is shortened due to a rise in CO, hypoxemia does not usually occur in healthy individuals, due to the recruitment and distension of capillaries and rise in alveolar oxygen. People with the lung disease mentioned above fail to recruit enough additional capillaries and exercise induced hypoxemia may occur (Sarkar et al., 2017). Also, healthy individuals can develop hypoxemia during exercise. Capillary transit time at rest is about 1.5 s and can drop to 0.42 s during exercise (Warren et al., 1991) leading to decreased oxygen saturation. Even healthy, highly trained endurance athletes may exhibit hypoxemia, either during moderate exercise by a relative hypoventilation induced by endurance training, or during high intensity exercise by ventilation/perfusion mismatch and/or diffusion limitation (Prefaut et al., 2000). Nevertheless, measurement of initial oxygen saturation in athletes with suggested pulmonary edema is recommended as a useful and practical test. Furthermore, if pathologic, it is a good tool for follow up monitoring and treatment control.

#### Radiological examination

The chest radiograph is one of the most practical and useful methods of visualization and quantifying pulmonary edema (Milne et al., 1985; Pistolesi et al., 1985). Mild pulmonary edema shows evidence of upper lobe diversion with constriction of lower lobe vessels and enlargement of upper lobe vessels, correspondent to a pulmonary capillary wedge pressure (PCWP) between 13 and 18 mmHg. Interstitial edema with loss of vascular definition, peribronchial cuffing, and Kerley lines occur with an elevation of PCWP to 19–25 mmHg. PCPW above 25 mmHg produces alveolar filling showing airspace opacities in the perihilar and lower lung zones (Ganter et al., 2006; Green and Klein, 2012). Moon et al. (2016b) showed that values of 18 ± 3.9 mmHg can be reached in SIPE-susceptible individuals by rapid submersion into water of 20°, rising to 18.9 ± 5.5 mmHg with additional exercise.

Pleural effusion can also occur (Cochard et al., 2005). 250–600 ml of fluid are required for it to be evident on an erect anteroposterior chest radiograph (Burgener et al., 2007). Smaller amounts about 50 ml could be detected in a lateral radiograph by blunting of the sharp posterior costophrenic angle. Ultrasound has a sensitivity of 100% by detecting pleural fluid as little as 5–50 ml. Computed tomography (CT) of the chest is not routinely used, but is very sensitive in detecting pulmonary edema and pleural effusion (Froudarakis, 2008). Signs of pulmonary edema are usually bilateral, but can be unilateral (Mahon et al., 2002).

In addition to visualization of possible pulmonary edema, other pathologies causing symptoms like malignancies, infections or some types of cardiac abnormalities with cardiac chamber enlargement can possibly be excluded with chest radiograph or chest CT.

In 34 of the 38 cases, a radiological examination (33 X-Ray, 1 CT-scan) was mentioned in the report, of which 24 (71%) showed signs of pulmonary edema. Ten (26%) had a normal initial chest X-Ray and in four cases (11%), radiological examination was not mentioned.

Finally, X-ray findings are subjective. Radiographic assessment of pulmonary edema shows fair to good reproducibility, but the clinical correlations seem to be modest (Worrell et al., 1996).

#### Therapy and recovery

The hallmark of SIPE is the rapid resolution of initial signs and symptoms within 48 h. The symptoms usually resolve after normalization of the physiologic environment by removal from water to a warm environment and by supportive treatment, sometimes even without any examination. Thirty-one of the thirty-eight cases (82%) mentioned a rapid resolution of the initial signs and symptoms, at least within 48 h. In 15 of these 31 cases with a rapid normalization (48%), *restitutio ad integrum* was confirmed with a normal follow up chest X-ray within 48 h. In two cases (5%), a follow up chest X-ray 2 days after onset was not totally normal and in five cases (13%), it was not clear when the normalization exactly occurred.

Administered therapies were reported in 35 of the 38 cases (92%) and consisted mostly of supplement oxygen therapy (27 cases, 77%): in 23% oxygen alone, in 37% in combination with β_2_-agonist and in 11% in combination with diuretics. Other therapies included continuous positive airway pressure (CPAP) (two cases), antibiotics (two cases), nitroglycerine (one case) and prednisolone (one case). Two cases resolved spontaneously without any intervention.

**Figure 1:**
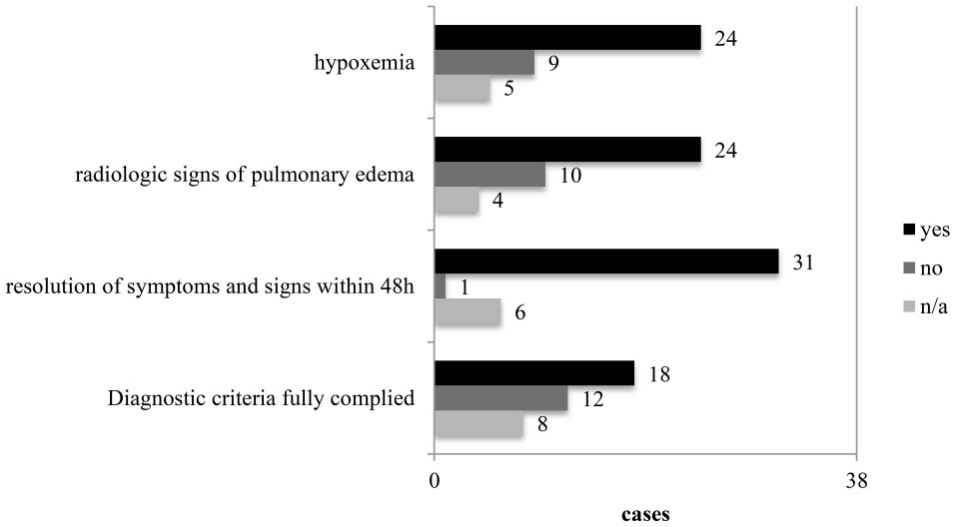
Diagnostic key points in the 38 cases.

In the 38 published cases (Table 1), we found remarkable differences in the diagnostic pathways mentioning the above discussed points (Figure 1). Based on our findings, we propose the checkpoint listed in Table 2 for improving the diagnosis and management of SIPE. As mentioned before, the pathophysiology of SIPE is complicated and a lot of different factors are involved in the development of this condition. The best confirmation of the diagnosis of SIPE is the complete resolution of symptoms of pulmonary edema within 48 h.

**Table 2:** Diagnostic checkpoints and management of SIPE.

History	Exercise in cold water
	Absence of water aspiration
	Absence of diseases concerning the cardiopulmonary system
	Acute onset of symptoms during or immediately after swimming
Symptoms	Cough and/or dyspnoea and/or chest tightness
	Haemoptysis
Clinical findings	Auscultation suggesting airway process (cracles, rales, wheezing)
Diagnostic testing	Hypoxemia^a^
	Radiological findings compatible with pulmonary edema
Management	Normalization of environment
	Supportive oxygen
	Occasionally β_2_-agonists
	Monitoring, occasionally follow up examinations

a *defined as arterial oxygen <60 mmHg or pulse oximeter values <90%, http://www.mayoclinic.org/symptoms/hypoxemia/basics/definition/sym-20050930*.

Recurrent episodes of SIPE often occur. In five of the 38 summarized cases (13%), one or more recurrent episodes are reported (Pons et al., 1995; Weiler-Ravell et al., 1995; Carter and Koehle, 2011). Adir et al. (2004) reported a recurrence rate of 22.9 %. Shupak et al. (2000) found a significantly higher recurrence rate after severe events of SIPE 75% of these severe events occurred after a previous mild or severe episode, supporting a certain degree of susceptibility of subjects suffering from SIPE. Medical staff caring for individuals with SIPE should be aware and brief their patients about the possibility of recurrence.

## Conclusions

Due to an increased recognition as a discrete diagnosis over the last years and the increase in participation in endurance competitions, the diagnosis of swimming induced pulmonary edema will play an essential role in the future, especially for medical teams taking care of the athletes in swimming events or sport events including a swimming section. The pathophysiology is not fully understood due to many different conditions that seem to play a role in the development of SIPE, making the diagnosis difficult. Nevertheless, SIPE can be assumed based on history, and clinical presentation. Diagnostic tests including monitoring of oxygen saturation and a radiological examination could support the diagnosis. The hallmark of SIPE is rapid resolution of initial signs and symptoms, in most cases within 48 h. Recurrent episodes often occur.

## Author contributions

HG collected all studies and drafted the manuscript, PN, RM, and BK helped in drafting the manuscript. All authors approved the final version.

### Conflict of interest statement

The authors declare that the research was conducted in the absence of any commercial or financial relationships that could be construed as a potential conflict of interest.
